# Overexpression of *AtPCS1* in tobacco increases arsenic and arsenic plus cadmium accumulation and detoxification

**DOI:** 10.1007/s00425-015-2428-8

**Published:** 2015-11-13

**Authors:** Letizia Zanella, Laura Fattorini, Patrizia Brunetti, Enrica Roccotiello, Laura Cornara, Simone D’Angeli, Federica Della Rovere, Maura Cardarelli, Maurizio Barbieri, Luigi Sanità di Toppi, Francesca Degola, Sylvia Lindberg, Maria Maddalena Altamura, Giuseppina Falasca

**Affiliations:** Department of Environmental Biology, “Sapienza” University of Rome, Rome, Italy; Dipartimento di Biologia e Biotecnologie Charles Darwin, “Sapienza” University of Rome, Rome, Italy; Dipartimento di Scienze della Terra, dell’Ambiente e della Vita, Polo Botanico Hanbury, University of Genoa, Genoa, Italy; Istituto di Biologia Medicina Molecolare e Nanobiotecnologie Consiglio Nazionale delle Ricerche, “Sapienza” University of Rome, Rome, Italy; Dipartimento di Scienze della Terra, “Sapienza” University of Rome, Rome, Italy; Department of Life Sciences, University of Parma, Parma, Italy; Department of Ecology, Environment and Plant Sciences, Stockholm University, Stockholm, Sweden

**Keywords:** Arsenic and cadmium, *AtPCS1* overexpression, Element analysis, Leaf crystal, *Nicotiana tabacum*, Phytochelatin, Root damage

## Abstract

**The heterologous expression of*AtPCS1*in tobacco plants exposed to arsenic plus cadmium enhances phytochelatin levels, root As/Cd accumulation and pollutants detoxification, but does not prevent root cyto-histological damages.**

High phytochelatin (PC) levels may be involved in accumulation and detoxification of both cadmium (Cd) and arsenic (As) in numerous plants. Although polluted environments are frequently characterized by As and Cd coexistence, how increased PC levels affect the adaptation of the entire plant and the response of its cells/tissues to a combined contamination by As and Cd needs investigation. Consequently, we analyzed tobacco seedlings overexpressing *Arabidopsis phytochelatin synthase1* gene (*AtPCS1*) exposed to As and/or Cd, to evaluate the levels of PCs and As/Cd, the cyto-histological modifications of the roots and the Cd/As leaf extrusion ability. When exposed to As and/or Cd the plants overexpressing *AtPCS1* showed higher PC levels, As plus Cd root accumulation, and detoxification ability than the non-overexpressing plants, but a blocked Cd-extrusion from the leaf trichomes. In all genotypes, As, and Cd in particular, damaged lateral root apices, enhancing cell-vacuolization, causing thinning and stretching of endodermis initial cells. Alterations also occurred in the primary structure region of the lateral roots, i.e., cell wall lignification in the external cortex, cell hypertrophy in the inner cortex, crushing of endodermis and stele, and nuclear hypertrophy. Altogether, As and/or Cd caused damage to the lateral roots (and not to the primary one), with such damage not counteracted by *AtPCS1* overexpression. The latter, however, positively affected accumulation and detoxification to both pollutants, highlighting that Cd/As accumulation and detoxification due to PCS1 activity do not reduce the cyto-histological damage.

**Electronic supplementary material:**

The online version of this article (doi:10.1007/s00425-015-2428-8) contains supplementary material, which is available to authorized users.

## Introduction

The semimetal arsenic (As) and the metal cadmium (Cd) are toxic to all forms of life. Due to their origin from both natural sources and human activities, these elements are present in soils all over the world, even at high levels. Arsenic is present in the environment in two main inorganic forms: arsenite [As(III)], prevalent species in reducing environments, and arsenate [As(V)], dominant species in aerobic soils (Zhao et al. 2009). Plants take up As mainly as As(V) and accumulate it preferentially in the roots. Arsenate, being an analogue of phosphate, is transported into plant cells via the phosphate transporters (Meharg and MacNair 1992), and negatively affects essential metabolic processes. Therefore, exposure to As(V) causes stress in plants, including inhibition of growth (Finnegan and Chen 2012 and references therein), and alteration of leaf gas-exchange, pigment content and water potential (Stoeva et al. 2005). Arsenate is easily reduced to As(III) in the cytosol (Meharg and Hartley-Whitaker 2002), either enzymatically or non-enzymatically (Verbruggen et al. 2009). Arsenite reacts with protein sulfhydryl groups (–SH), inhibiting cellular functions and even causing death (Ullrich-Eberius et al. 1989). Cadmium is among the most toxic elements to living organisms. It enters the food chain through plants that take up it very easily. Cadmium alters a large number of physiological processes of the plants (Sanità di Toppi and Gabbrielli 1999).

Polluted environments frequently show the simultaneous existence of different toxic elements, e.g. Cd, As, Cu, Pb, Cr and Zn (Kim et al. 2003; Loska et al. 2004). Many plants have evolved sophisticated and complex mechanisms to activate metal/semimetal detoxification, such as immobilization, chelation and compartmentalization, and extrusion by leaf trichomes (Choi et al. 2001; Clemens et al. 2002; Gasic and Korban 2007; Tuli et al. 2010). Moreover, remodelling the root architecture, e.g. enhancing lateral root formation, is a wide used strategy to face a multi-polluted context (Sofo et al. 2013). However, information on the effects of combined contamination on plant uptake, growth and related cyto-histological events is still reduced. Cadmium and As may affect each other’s uptake, as well as that of essential elements (Carbonell-Barrachina et al. 1995; Das et al. 1997). Both enter the plant by the roots using the transporters of the essential nutrients. Arsenate enters by the Pi transport system, arsenite by the NIP subfamily transporters of aquaporins, and cadmium by the Ca^2+^, Fe^2+^, Mn^2+^ and Zn^2+^ transporters (Verbruggen et al. 2009, and references therein). Moreover, both Cd and As injure cell structures by inhibiting the activity of numerous enzymes, showing a similar behaviour toward the SH groups of the biological molecules and a similar sequestration machinery (dos Santos et al. 2012).

A major plant defence mechanism to detoxify metal/semimetal ions is to complex them with metal-binding thiol-peptides, such as phytochelatins (PCs) (Cobbett 2000; Sanità di Toppi et al. 2003). Phytochelatins are cysteine-rich peptides that chelate, by means of thiol groups, various toxic metals and semimetals (Liu et al. 2010). Phytochelatins [general structure (*γ*-Glu-Cys)nGly] are synthesized from reduced glutathione (GSH) in a reaction catalyzed by the enzyme phytochelatin synthase (PCS), a *γ-*glutamylcysteine dipeptidyl transpeptidase (EC 2.3.2.15) (Cobbett 2000; Sanità di Toppi et al. 2003). It was demonstrated that higher plants, algae, some fungi, lichens, bryophytes, and even some animal species produce PCs in response to semimetal/metal stress (Vatamaniuk et al. 2001; Pawlik-Skowrońska et al. 2002, 2007; Rea 2012; Petraglia et al. 2014). The *PCS* gene is constitutively expressed in plant cells and is activated in the presence of metals/semimetals, including Cd and the two main inorganic As species (Grill et al. 1989; Schmöger et al. 2000). It has been isolated, characterized and overexpressed in numerous species (Lee et al. 2003; Li et al. 2004; Gasic and Korban 2007; Brunetti et al. 2011) to increase PC levels and metal/semimetal accumulation, and detoxification. *PCS* genes coming from various species were overexpressed in *Nicotiana tabacum* (Pomponi et al. 2006; Wojas et al. 2008, 2010a, b; Liu et al. 2011; Shukla et al. 2012). Moreover, different constructs and experimental conditions, including exposure to various concentrations and forms of toxic elements, have been used, resulting in changes in the metal responses of the transgenic plants.

*Nicotiana tabacum* is one of the most socially and economically important crops worldwide. It is not a hyperaccumulator of semimetals/metals although it is able to take and accumulate them at consistent level (Zvobgo et al. 2015). In addition, it is a good candidate for phytoextraction because of its high biomass, moderate soil chemical/physical requirement, fast growth rate and easy to harvesting (Sarret et al. 2006). Moreover, *PCS* genes coming from *Arabidopsis thaliana* (*AtPCS1*), *Caenorhabditis elegans* (*CePCS*) and from *Ceratophyllum demersum* (*CdPCS1*) were successfully overexpressed in tobacco plants (Pomponi et al. 2006; Wojas et al. 2008, 2010a, b; Shukla et al. 2012). Previous results on the overexpression of *AtPCS1* in tobacco and *Arabidopsis* have shown an increased production of PCs, with a general increment of plant Cd-detoxification when the metal was supplied at specific concentrations (Pomponi et al. 2006; Brunetti et al. 2011). However, in tobacco overexpressing *AtPCS1* gene, an exogenous application of GSH resulted into enhanced Cd detoxification (Pomponi et al. 2006). Nowadays, there is no information about the mechanisms of semimetal/metals accumulation and detoxification in tobacco overexpressing *PCS* genes exposed to As and Cd simultaneously.

In tobacco leaves, two types of glandular trichomes, i.e., the short trichomes, with multicellular heads, and long trichomes, are present (Meyberg et al. 1991). Whereas the latter ones serve as a defence against insect attack because of their secretion of useful natural products (McCaskill and Croteau 1999), the short trichomes can exude Cd ions via a Ca-Cd crystallization (Choi et al. 2001; Choi and Harada 2005; Sarret et al. 2006). Leaf trichomes are involved in the detoxification of metal/semimetal also in other species, such as *Brassica juncea* (Salt et al. 1995), *Alyssum lesbiacum* (Krämer et al. 1997), *Arabidopsis halleri* and *Vigna radiata* (Küpper et al. 2000; Gupta and Bhatnagar 2015). The capability of tobacco trichomes to extrude crystals containing As and/or Cd in plants simultaneously exposed to the metal and the semimetal has not yet been investigated, as well as a possible interference of a higher PC level, obtained by *AtPCS1* overexpression, on the As/Cd detoxification by leaf crystals.

Thus, the goal of this work was to investigate As accumulation in the presence or absence of Cd and detoxification in tobacco plants overexpressing *AtPCS1* gene, and the possible cyto-histological consequences. In order to pursue this aim, tobacco seedlings were exposed to As and Cd, supplied both alone and in combination, and the semimetal/metal levels, thiol-peptides concentrations, cyto-histological root modifications, and Cd/As extrusion by leaf trichomes were evaluated. Results show that *AtPCS1* overexpression in tobacco promotes accumulation and detoxification of Cd and As, but does not prevent root cyto-histological damage.

## Materials and methods

### Plant materials and growth analysis

Seeds of a *Nicotiana tabacumrolB*-*AtPCS1* line, selected for high PC levels and Cd accumulation according to Pomponi et al. (2006) were used in the present work. Seeds of *N. tabacum* cv. Petit Havana SR1 (Maliga et al. 1973), and of *N. tabacumrolB*, i.e., the genetic background of *rolB*-*AtPCS1,* were used as controls. All seeds came from the authors’ laboratory collection. Seeds were sterilized and sown on Petri dishes containing half-strength salts of MS solution (Murashige and Skoog 1962), 3 % sucrose and 0.8 % agar (i.e. MS medium). The Petri dishes were kept in an illuminated (16 h light/8 h dark photoperiod) thermostatic greenhouse at 21 °C and 70 % humidity. All the experiments for As and/or Cd exposure were carried out on 10-day-old seedlings transferred onto either refreshed MS medium or Hoagland medium, as specified below, supplemented or not (Control treatment) with either 50 or 200 μM Na_2_HAsO_4_.7H_2_O (i.e., 50 As and 200 As, respectively), or 60 μM CdSO_4_ (i.e., 60 Cd), or with 50 μM Na_2_HAsO_4_.7H_2_O plus 60 μM CdSO_4_ (i.e., 50 As + 60 Cd). The As concentration in the latter treatment was selected because preliminary experiments had shown that the higher As concentrations, when combined with Cd, induced extensive damage to the entire plants.

### Morphological analyses

Thirty seedlings per genotype and treatment were transferred onto refreshed MS medium containing or not As and/or Cd, harvested after 9 and 16 days, and immediately weighed. Other 30 seedlings per genotype and treatment were transferred into square plates containing the refreshed MS medium supplemented or not with the selected As and/or Cd concentrations, and placed in vertical position. The primary root (PR) length was measured after 9 days. PR length evaluation was not repeated at day 16 because the excessive development of the roots in the controls at this day did not allow a correct PR analysis.

### RT-PCR analysis

Total RNA was extracted from wild type, *rolB* and *rolB*-*AtPCS1* seedlings grown for 16 days onto MS medium, in the absence of CdSO_4_ as described in Cecchetti et al. (2004). Reverse transcriptase reactions were performed as previously described (Brunetti et al. 2011). RT-PCR products were obtained after 30 PCR cycles. The primers for *AtPCS1* were 5′-TCAAGTATCCCCCTCACTGG-3′and 5′-TTTGCGTCGATGGCACTAAC-3′and for the actin gene 5′-CTTGCACCAAGCAGCATGAA-3′ and 5′-CCGATCCAGACACTGTACTTCCTT-3′.

### Thiol peptide analysis

After 9 and 16 days of metal/semimetal exposure on refreshed MS medium, seedlings (200 mg FW per genotype, treatment and data point) were harvested and immediately frozen in liquid nitrogen either as whole plants or roots and shoots separately. GSH and phytochelatin quali-quantitative determinations were carried out by post-column derivatization HPLC according to Pomponi et al. (2006).

### Elements determination

To analyze As and Cd levels in plant organs two different experiments were carried out. In the first experiment, 200 seedlings per genotype were transferred to hydroponic culture on liquid Hoagland medium containing 0.2 % of Hoagland salts (Hoagland and Arnon 1938) to allow their acclimatization to this new culture condition. After 7 days, the seedlings were transferred to refreshed Hoagland medium supplemented or not with either 50 As and 200 As or 60 Cd, or with 50 As + 60 Cd under continuous shaking. Fifteen plants per genotype and treatment were taken up after 9 and 16 days, and thoroughly washed with deionised water. Shoots and roots were then isolated and dried at 60 °C for two days.

In the second experiment, other 100 seedlings per genotype were transferred on refreshed MS solid medium supplemented or not with either 50 As and 200 As or 60 Cd, or with 50 As + 60 Cd and cultured for 9 days. Fifteen plants per genotype and treatment were thoroughly washed with deionised water. Shoots and roots were isolated and dried at 60 °C for two days. Dried tissues, coming from both experiments, were weighed, ground in a mortar and treated with 65 % nitric acid (5 ml) in 25 ml glass flasks fitted with air-cooled condensers. Solutions were heated at 140 °C for 3 h. Cd and As concentration was measured by Inductively Coupled Plasma Mass Spectrometry (ICP-MS), using a Thermo Scientific X Series 2 spectrometer equipped with CETAC AT5000. This instrument operates in the helium collision cell mode to eliminate interference from isobaric polyatomic species via kinetic energy discrimination. The instrument was standardized with multi-element standards containing Cd and As at concentrations of approximately 0.1, 0.5, 1.0, and 10 ng/ml in matrix of 2 % HNO_3_. For trace level analysis, the accuracy of the analytical method was assessed during a Certified Reference Material (CRM).

### Intracellular Cd detection

Intracellular Cd localization was performed by Leadmium^TM^ Green AM (Molecular Probes, Invitrogen, Calsbad, CA, USA) fluorescent dye. After 16 days of treatments on refreshed MS medium with either 60 Cd or 50 As + 60 Cd, leaf and root protoplasts were extracted. The enzymatic digestion for leaf and root protoplasts was carried out according to Lindberg et al. (2004) and (2007), respectively. Leaf and root protoplasts were loaded with Leadmium Green AM fluorochrome. This fluorochrome complexes Cd^2+^, however it could also complex lead and calcium when present at high levels (Molecular Probes, Invitrogen). A stock solution of Leadmium Green AM dye was prepared by solving 50 μg of the fluorochrome in 50 μl of dimethyl sulfoxide (DMSO). Protoplasts were purified and incubated with 5 μl of Leadmium Green solution in 1 ml of a medium containing 0.1 mM CaCl_2_, 0.5 M sorbitol, 0.05 % w/v polyvinyl-pirrolidone10, 0.2 % w/v bovine serum albumin, and 5 mM TRIS/MES (pH 5.5) at 4 °C for 1 h in the dark. Then the solution was centrifuged and the pellet re-suspended in the same refreshed medium at pH 7. Before microscopic observations, the protoplasts were stored at room temperature for 30 min in the dark. The fluorescence was observed using a LEICA DMRB microscope equipped with a specific filter sets (EX BP 484–15 nm and LP 517–30 nm). The images were acquired with a Leica DC500 digital camera and analyzed with a personal computer (Opti-Xex GX 240 MT) using the Leica IM1000 image-analysis software (Leica). Cell fluorescent regions were selected from 30 single protoplast images per genotype and treatment under constant light conditions, and the mean intensity value of epifluorescence quantified using ImageJ (National Institute of Health, Bellevue, WA, USA) software, and expressed in arbitrary units (AUs, from 0 to 255).

### Histological analysis

The histological analyses were carried out on ten lateral roots (LRs) randomly sampled among 30 plants per genotype and treatment after 9 days of As and/or Cd exposure on refreshed MS medium. About 1–1.5 cm of LR (i.e., from root cap to primary structure zone) were excised, fixed for 24 h in 70 % ethanol, soon after dehydrated, embedded in Technovit 7100 (Heraeus Kulzer), sectioned at 4 μm with an automatic microtome (Microm HM 350 SV), stained with 0.05 % (w/v) toluidine blue, and examined under the Leica DMRB microscope. The sections were also observed under a microscope equipped with a specific filter sets (EX BP 340–380 nm, LP 425 nm) suitable for lignin autofluorescence detection. All the histological and morphological images were acquired with a DC500 video camera applied to the microscope.

### Microanalysis of leaf trichomes

Fifteen seedlings per genotype and treatment were transferred into Magenta™ vessels (Sigma) containing refreshed MS medium supplemented with or without 60 Cd or 50 As + 60 Cd. After 2, 5, 6 and 8 weeks, three leaves per plant were cut, mounted on aluminum stubs, and examined by variable pressure scanning electron microscopy (SEM) with a LEO 1450 VP microscope (Zeiss, Oberkochen, Germany) at an acceleration voltage of 30 kV. Microanalyses were done with the EDS INCA 300 (OXFORD Instruments). Glandular trichomes distributed on the leaf *s*urfaces were observed. Distribution and content of elements in the trichomes and extruded crystals were examined, according to Choi et al. (2001).

### Statistical analysis

Statistical analysis was performed using ANOVA test (one way and two way) followed by Tukey test through GraphPad Prism 6 software. All the experiments were performed in triplicate with similar results.

## Results

### Overexpression of *AtPCS1* in tobacco increases As and As plus Cd detoxification

The expression of *AtPCS1* in tobacco *rolB*-*AtPCS1* plants was confirmed by RT-PCR analysis (Fig. S1).

**Fig. 1:**
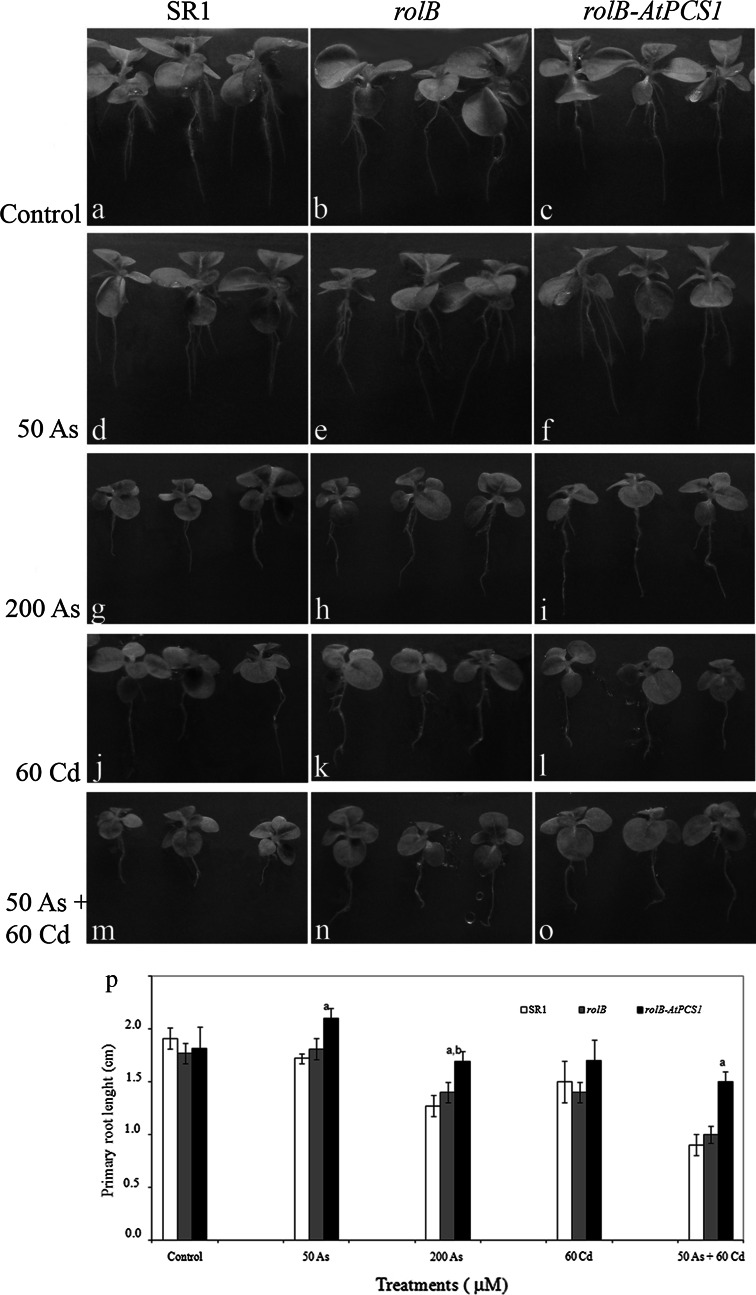
Phenotype of wild-type (SR1, **a**, **d**, **g**, **j**, **m**) and transgenic (*rolB*, **b**, **e**, **h**, **k**, **n**, and *rolB*-*AtPCS1*, **c**, **f**, **i**, **l,** **o**) tobacco plants non-treated (Control, **a**–**c**) and treated with either 50 or 200 μM Na_2_HAsO_4_.7H_2_O (50 As, **d**–**f** and 200 As, **g**–**i**), or with 60 μM CdSO_4_ (60 Cd, **j**–**l**), or with 50 μM Na_2_HAsO_4_.7H_2_O plus 60 μM CdSO_4_ (50 As + 60 Cd, **m**–**o**), and mean length (±SE) of primary roots after 9 days of treatment (**p**). *Letter a*, *P* < 0.01 difference with respect to the other genotypes within the same treatment. *Letter b*, *P* < 0.05 difference with respect to the *rolB* within the same treatment. Columns followed by *no letter* within the same treatment are not significantly different. Significant differences between treatments are reported in the text. *n* = 30

**Fig. 2:**
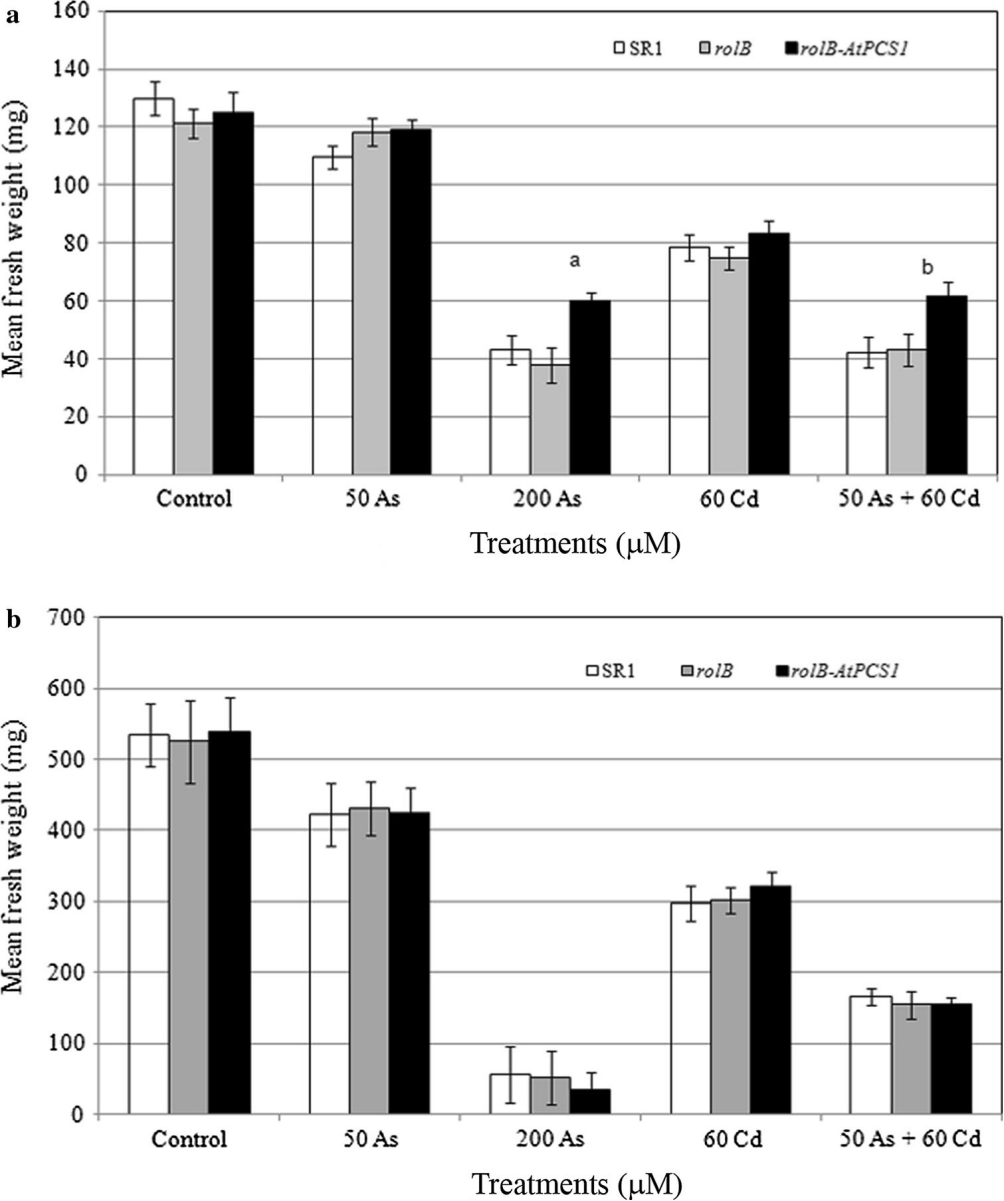
Mean fresh weight (±SE) of SR1, *rolB* and *rolB*-*AtPCS1* plants after 9 (**a**) and 16 (**b**) days of treatment without Cd and As (Control), and with 50 μM Na_2_HAsO_4_.7H_2_O (50 As) or 200 μM Na_2_HAsO_4_.7H_2_O (200 As), or 60 μM CdSO_4_ (60 Cd), or 50 μM Na_2_HAsO_4_.7H_2_O plus 60 μM CdSO_4_ (50 As + 60 Cd). *Letter a*, *P* < 0.01 difference with the other genotypes within the same treatment. *Letter b*, *P* < 0.05 difference with the other genotypes within the same treatment. Columns followed by *no letter* within the same treatment are not significantly different. *n* = 30

To evaluate As and As plus Cd detoxification in tobacco overexpressing *AtPCS1* plants, primary root (PR) length and fresh weight (FW) in SR1, *rolB* and *rolB*-*AtPCS1* plants were measured. In the absence of the semimetal and/or the metal (i.e., Control treatment), both PR length and FW of transgenic plants (i.e., *rolB* and *rolB*-*AtPCS1*) were comparable to those of the wild type (i.e., SR1) (Figs. 1a–c, p, 2a, b). The exposure to 50 As for 9 days did not modify either the PR length or the mean FW with respect to the Control treatment within each genotype (Figs. 1d–f, a–c, in comparison, 2a); however, the PRs of *rolB*-*AtPCS1* plants were significantly longer than SR1 ones (Fig. 1p). Plant growth was strongly inhibited by the exposure to 200 As (Figs. 1g–i, 2a). Nevertheless, *rolB*-*AtPCS1* plants showed the PR and the FW significantly less reduced in comparison with SR1 and *rolB* (Figs. 1p, 2a). The presence of 60 Cd reduced the PR growth and FW in a similar manner in all genotypes with respect to Control treatment (Figs. 1j–l, p, 2a), supporting the results on PR growth previously found by Pomponi and co-workers (Pomponi et al. 2006). When the plants were exposed to 50 As + 60 Cd, both PR length and FW were reduced in comparison with the Control treatment, but the reduction was significantly lower in *rolB*-*AtPCS1* in comparison with SR1 and *rolB* (Figs. 1m–o, p, 2a).

When the exposure to As and/or Cd lasted 16 days, a FW reduction was observed in all genotypes with respect to Control treatment. The reduction was more drastic in the presence of 200 As and 50 As + 60 Cd (Fig. 2b).

### Overexpression of *AtPCS1* induces high levels of PCs in plants treated with As in the presence/absence of Cd

**Fig. 3:**
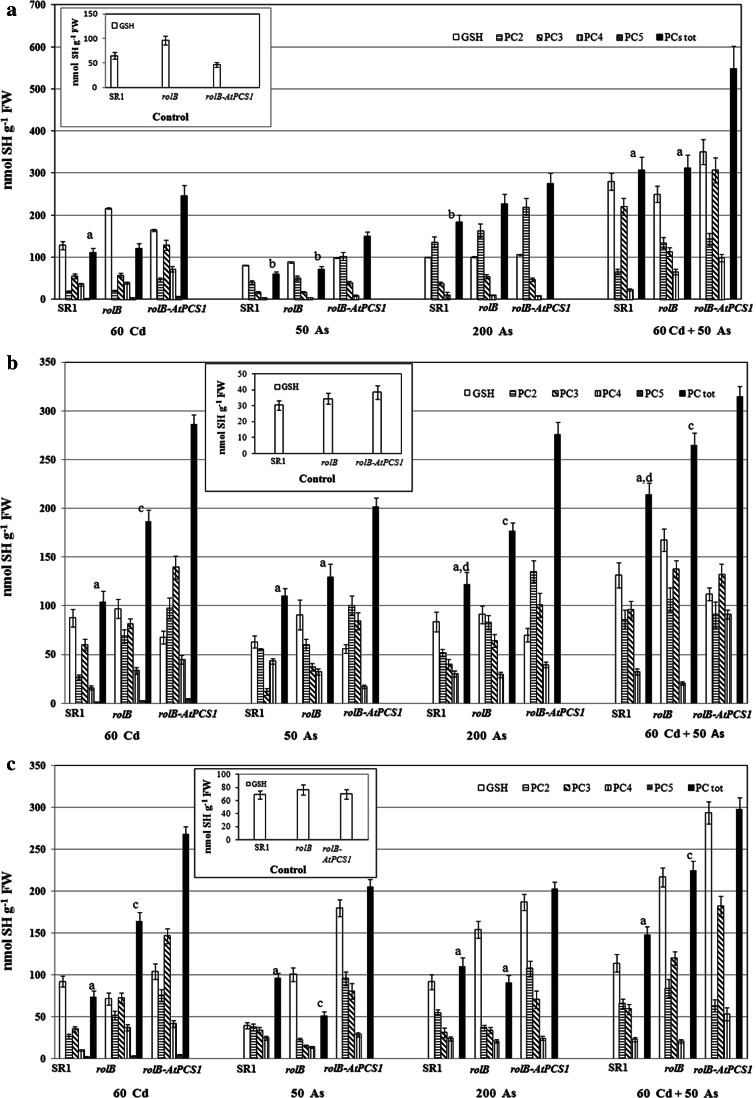
Mean levels of PCs (PC2, PC3, PC4, PC5 fractions and total PCs) and of endogenous GSH (±SE) in SR1, *rolB* and *rolB*-*AtPCS1* whole plants (**a**), roots (**b**) and leaves (**c**) of plants grown without Cd and As (Control, *insets*) or treated for 9 days with either 50 or 200 μM Na_2_HAsO_4_.7H_2_O (50 As and 200 As, respectively), or 60 μM CdSO_4_ (60 Cd), or with 50 μM Na_2_HAsO_4_.7H_2_O plus 60 μM CdSO_4_ (50 As + 60 Cd). *Letter a*, *P* < 0.01 difference in the total PCs within the same treatment. *Letter b*, *P* < 0.05 difference in the total PCs with *rolB*-*AtPCS1* within the same treatment. *Letter c*, *P* < 0.01 difference in the total PCs with *rolB*-*AtPCS1* within the same treatment. *Letter d*, *P* < 0.05 difference in the total PCs with SR1 within the same treatment. Columns of total PCs followed by the *same letter*/*no letter*, within the same treatment, are not significantly different. Significant differences between different PC fractions and between treatments are reported in the text. Means of three replicates

SR1, *rolB* and *rolB*-*AtPCS1* plants grown for 9 and 16 days in the absence (i.e., Control treatment) and in the presence of either 50 or 200 As, or 60 Cd or 50 As + 60 Cd were analyzed by HPLC to assess the effects of *AtPCS1* overexpression on endogenous GSH and PC production. In the Control treatment PCs (i.e., the PC2, PC3, PC4 and PC5 oligomers) were not detected, and the GSH content was similar, and stable over time, in all genotypes, either in whole plants and in roots and shoots separately (Fig. 3, Fig. S2, insets).

After 9 days of semimetal/metal treatment, GSH level significantly increased (*P* < 0.01) in all genotypes, and mainly in the presence of 50 As + 60 Cd (Fig. 3a–c). The production of PCs was induced in all genotypes, and at higher levels in *rolB*-*AtPCS1* plants (Fig. 3a–c), but with differences in PC fractions among treatments. In fact, in *rolB*-*AtPCS1* plants, PC3 was the most abundant fraction in the presence of Cd and As + Cd, whereas PC2 was the main fraction in the treatments with As alone. In all genotypes the PC5 fraction was detected only in the presence of 60 Cd alone, and at very low levels (Fig. 3a–c). The PC levels in the roots were slightly higher than in the aerial organs (Fig. 3b, c). However, the trend of total PCs and of the single oligomers was similar in whole plants and in both roots and shoots (Fig. 3a–c).

After 16 days, an increase in total PCs was observed in whole plants of all genotypes exposed to As and/or Cd (Fig. S2). The PCs trend was similar to that observed after 9 days, but with levels comparatively higher. *rolB*-*AtPCS1* plants showed a significantly higher content of total PCs compared to SR1 and *rolB* in all treatments (Fig. S2). PC5 levels increased consistently in all genotypes in comparison with day 9, but again only in the presence of 60 Cd alone (Fig. S2).

### Overexpression of *AtPCS1* leads to a higher accumulation of As in the roots

**Fig. 4:**
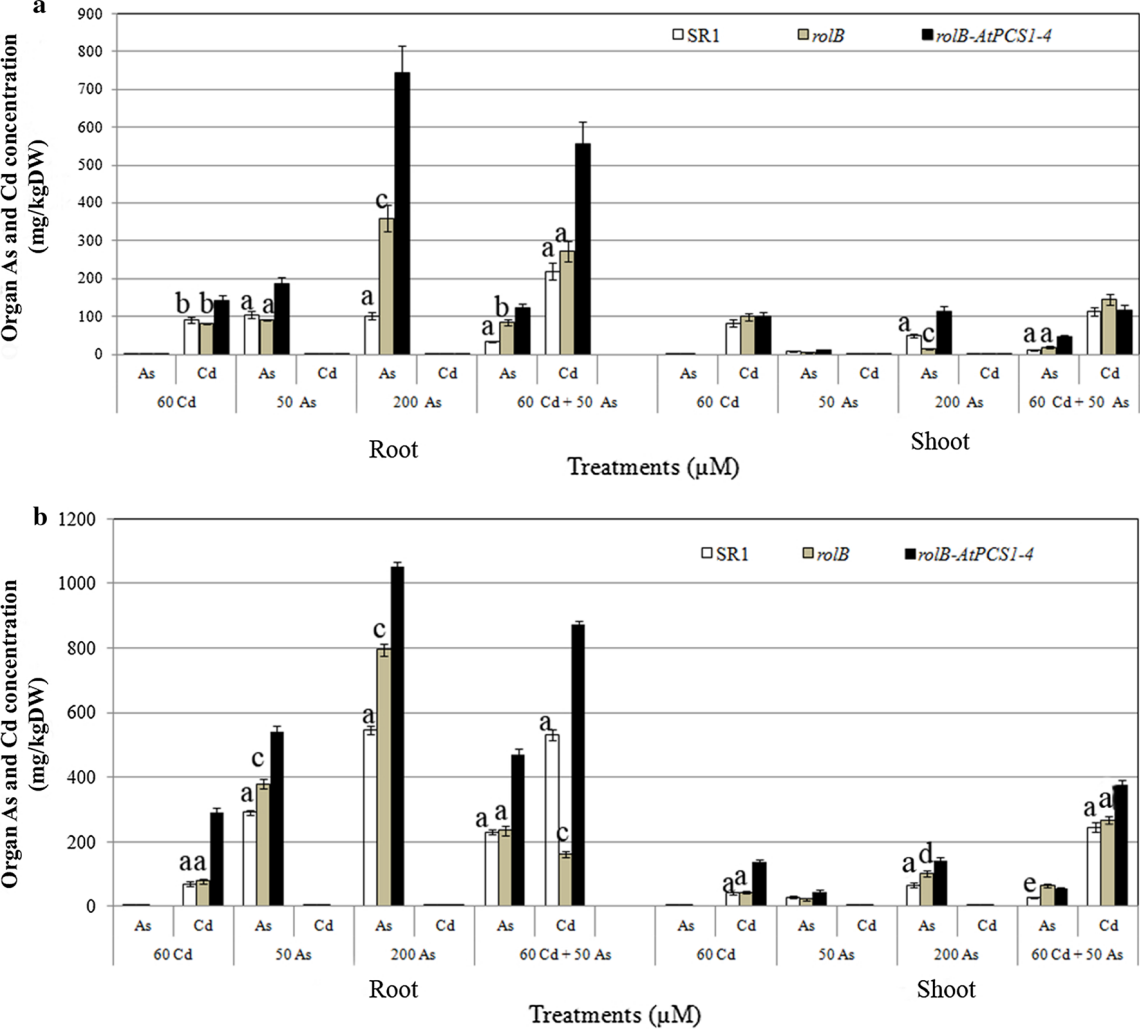
Mean concentrations (±SE) of As and Cd in roots and shoots of SR1, *rolB* and *rolB*-*AtPCS1* plants treated for 9 days with either 50 or 200 μM Na_2_HAsO_4_.7H_2_O (50 As and 200 As, respectively), or 60 μM CdSO_4_ (60 Cd), or with 50 μM Na_2_HAsO_4_.7H_2_O plus 60 μM CdSO_4_ (50 As + 60 Cd). Plants cultured either on Hoagland liquid medium (**a**) or on MS solid medium (**b**) after the 10 days growth on MS germination medium. *Letter a*, *P* < 0.01 difference within the same treatment. *Letter b*, *P* < 0.05 difference with *rolB*-*AtPCS1* within the same treatment. *Letter c*, *P* < 0.01 difference with *rolB*-*AtPCS1* within the same treatment. *Letter d*, *P* < 0.05 difference within the same treatment. *Letter e*, *P* < 0.05 difference with *rolB* within the same treatment. Columns followed by the *same letter*/*no letter* within the same treatment are not significantly different. Significant differences between treatments are reported in the text. Means of three replicates

To evaluate whether *AtPCS1* overexpression induces a higher accumulation of As in tobacco organs, and whether Cd presence combined with As modifies As accumulation, an ICP-MS analysis was carried out on roots and shoots of plants exposed/unexposed to 50 As, 200 As, 60 Cd, and 50 As + 60 Cd for 9 and 16 days. The analysis was carried out both on plants cultured on MS solid medium followed by Hoagland liquid medium and on plants cultured on MS solid medium for the entire cultural period. Control plants showed negligible levels of As and Cd after both 9 and 16 days, which were interpreted as a result of the instrument calibration (Fig. S3a–c). In both experimental conditions, the roots of *rolB*-*AtPCS1* plants treated for 9 days with As alone (50 and 200 µM) showed semimetal levels significantly higher than the roots of SR1 and *rolB* plants (Fig. 4a, b). Also Cd was accumulated in the roots of *rolB*-*AtPCS1* treated with Cd alone at significantly higher levels than in SR1 and *rolB* plants (Fig. 4a, b). The presence of both pollutants induced a higher Cd accumulation in the roots of all genotypes (*P* < 0.01), particularly in *rolB*-*AtPCS1,* compared to Cd alone treatment. By contrast Cd negatively affected As accumulation in comparison with treatments with As alone (*P* < 0.01 and *P* < 0.05, differences with both 50 and 200 As treatments, respectively; Fig. 4a, b). The levels of As and Cd in the shoots of all genotypes were in general lower than in the roots, independently on the treatment and the cultural condition (Fig. 4a, b). However, the As levels in the shoots of *rolB*-*AtPCS1* cultured on MS followed by Hoagland and treated with As + Cd, and 200 As, in particular, were significantly higher than in the other genotypes (Fig. 4a). Differently, in the shoot of *rolB*-*AtPCS1* cultured on MS medium alone, the As levels were higher than in the other genotypes only in the 200 As treatment (Fig. 4b). Cadmium accumulation in the leaves of *rolB*-*AtPCS1* plants, cultured on MS medium alone, was higher than in the other genotypes both in the treatment with Cd only or in the combined treatment (Fig. 4b).

After 16 days of treatment, the trend of As and Cd accumulation in roots and shoots was similar to that observed at day 9, although with highly enhanced levels (Fig. S4). Moreover, the three genotypes showed a poor ability to translocate the two pollutants to the shoot. The *rolB*-*AtPCS1* genotype continued to be the best genotype for As and Cd accumulation in the roots. Again the As plus Cd treatment caused a reduction of As accumulation in *rolB*-*AtPCS1* roots, and, by contrast, increased Cd accumulation (Fig. S4).

### Overexpression of *AtPCS1* enhances Cd compartmentalization in the vacuole of root and leaf cells, but the presence of As reduces it

To verify whether the higher accumulation of Cd in *rolB*-*AtPCS1* plants, exposed to Cd alone or Cd combined with As (Fig. 4), was related to a specific cellular compartmentalization of the metal, a fluorescence analysis using the selective Cd-sensing fluorochrome Leadmium™ Green AM dye was carried out in leaf and root protoplasts of SR1, *rolB* and *rolB*-*PCS1* plants cultured for 16 days on refreshed MS medium.

**Fig. 5:**
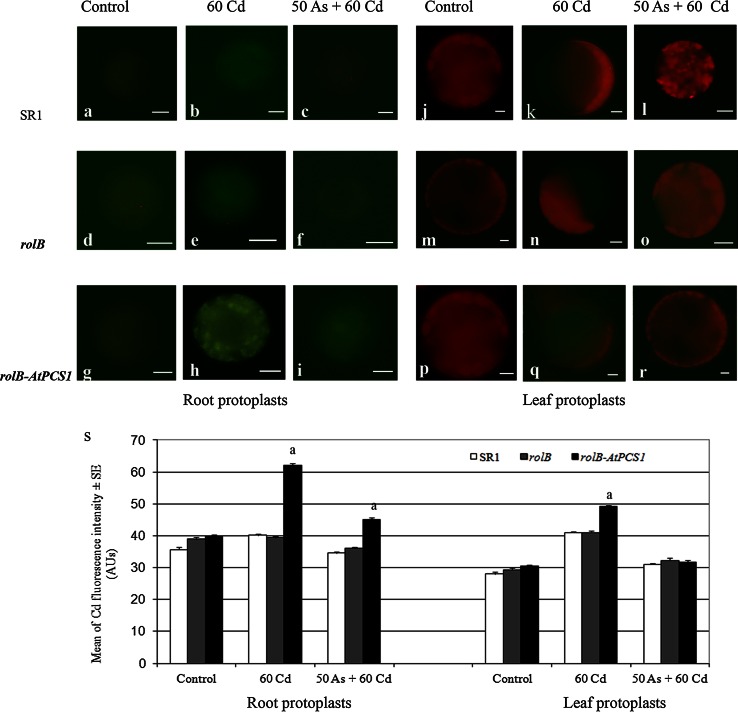
Detection of vacuolar Cd in SR1, *rolB* and *rolB*-*AtPCS1* root and leaf protoplasts. Protoplasts from plants cultured for 16 days without Cd and As (Control) or in the presence of 60 μM CdSO_4_ (60 Cd) or 50 μM Na_2_HAsO_4_.7H_2_O plus 60 μM CdSO_4_ (50 As + 60 Cd) were loaded with the Cd-sensitive probe Leadmium™ Green AM dye. **a**–**i** Fluorescent images of root protoplasts. **j**–**r** Fluorescent images of leaf protoplasts. **s** Mean Cd fluorescence intensity (±SE) in the vacuoles of root and leaf protoplasts measured using ImageJ 1.36b software and expressed in arbitrary units (AUs). *Letter a*, *P* < 0.01 difference within the same treatment. Columns followed by the *same letter/no letter* are not significantly different. Significant differences between treatments are reported in the text. *n* = 30. *Bars* 10 µm

Firstly, the protoplasts isolated from all genotypes of the Control treatment were analysed, and showed a similar low green fluorescence signal in the vacuole, hardly visible in the fluorescence images (Fig. 5a, d, g, j, m, p), but detectable by image software (Fig. 5s). Because other ions may affect the Leadmium™ Green AM fluorescence signal, its presence in the Control treatment (Fig. 5s) was interpreted as the result of the interaction between the fluorochrome and Ca^2+^, that naturally occurs in the cells, and is present in the culture medium, and in the protoplast extraction solutions (Lu et al. 2008). Moreover, the leaf protoplasts exhibited a red fluorescence signal due to chlorophyll autofluorescence (Fig. 5j–r). The results showed that the protoplasts extracted by roots and leaves of *rolB*-*AtPCS1* plants exhibited the highest green fluorescence signal in comparison with those from SR1 and *rolB* plants, except for the leaf protoplasts exposed to Cd + As. No significant difference occurred between the fluorescence of SR1 and *rolB* protoplasts (Fig. 5h, q, s). Moreover, in *rolB*-*AtPCS1* plants only, the presence of As combined with Cd reduced significantly (*P* < 0.01) the green fluorescence signal, showing a decreased Cd accumulation in the vacuole of root and leaf protoplasts in comparison with the Cd-alone treatment (Fig. 5i, r, s). However, in the vacuoles of the root protoplasts of *rolB*-*AtPCS1* the level of the fluorescence signal remained significantly higher than in the other two genotypes (Fig. 5s).

### Arsenic and cadmium induce the same cyto-histological alterations in tobacco roots independently of *AtPCS1* overexpression

During the first 10 days after germination, the seedlings grew without any exposure to semimetal/metal (see “Materials and methods”), allowing the regular development of all PR tissues, including the pericycle, i.e., the tissue responsible for the initiation of the lateral roots (LRs). For this reason, the histological structure of the PR during the period of exposure to the pollutants was excluded from the analysis, whereas the LRs were analyzed.

**Fig. 6:**
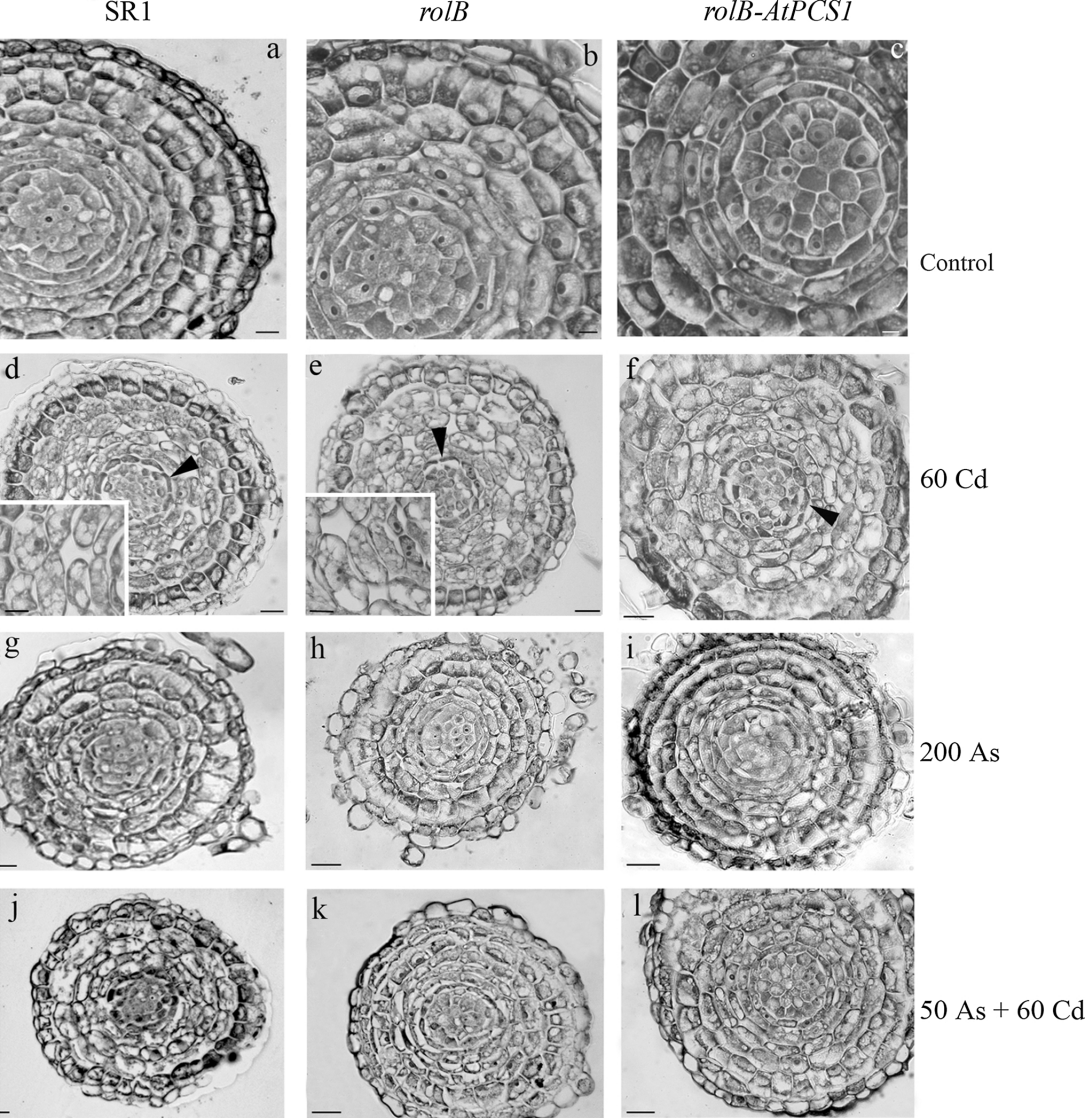
Histological analysis of the apical meristematic region of lateral roots in SR1, *rolB* and *rolB*-*AtPCS1* after 9 days of treatment. **a**–**c** Regular structure in roots of plants grown without Cd and As (Control). **d**–**f** Vacuolation in the ground meristem forming the cortex, as magnified in the *insets*, and thinning of the still meristematic endodermis (*arrowheads*) of roots exposed to 60 μM CdSO_4_ (60 Cd). **g**–**i** Almost regular structure of roots exposed to 200 μM Na_2_HAsO_4_.7H_2_O (200 As). **j**–**l** Vacuolization in the forming cortex of roots exposed to 50 μM Na_2_HAsO_4_.7H_2_O plus 60 μM CdSO_4_ (50 As + 60 Cd). Transverse sections. *Bars* 20 µm (**a**–**c** and *insets* in **d**, **f**), 30 µm (**d**–**l**)

**Fig. 7:**
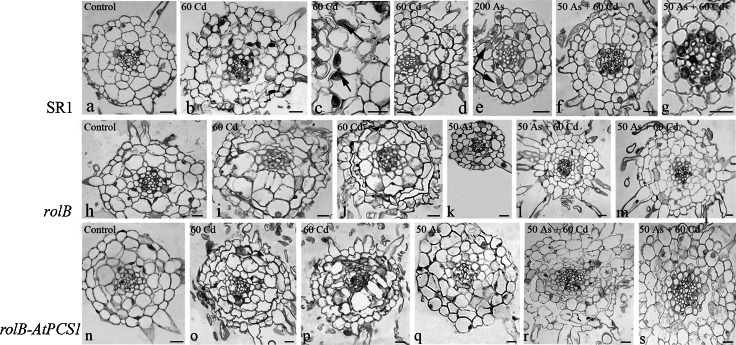
Histological analysis of primary structure region of lateral roots of SR1 (**a**–**g**), *rolB* (**h**–**m**) and *rolB*-*AtPCS1* (**n**–**s**) after 9 days of treatment on refreshed MS medium. **a**, **h**, **n** Root non-treated with the semimetal/metal showing their regular structure. **b**–**d**, **i**–**j**, **o**–**p** Roots of the three genotypes exposed to 60 μM CdSO_4_ showing cellular (**b**, **i**–**j,** **o**–**p**) and nuclear (**c**, *arrows*, **i**) hypertrophy in the cortical cells, and increased hair formation (**d**, **j**, **o**–**p**). **e**, **k**, **q** Roots of the three genotypes exposed to 200 (**e**) and 50 (**k**, **q**) μM Na_2_HAsO_4_.7H_2_O showing a regular structure except for rare hypertrophic nuclei in the cortex (*arrows* in **e**). **f**–**g**, **l**–**m**, **r**–**s** Roots of the three genotypes exposed to 50 μM Na_2_HAsO_4_.7H_2_O plus 60 μM CdSO_4_ showing cortical cell hypertrophy (**f**, **m**, **r**–**s**). Transverse sections. *Bars* 30 µm

To determine the cyto-histological alterations due to As and/or Cd exposure in LRs, their meristematic apical zone and primary differentiation zone were analyzed in the absence (i.e., Control treatment) or presence of 50 As, 200 As, 60 Cd, and 50 As + 60 Cd, at day 9, i.e. when PR length and seedling FW had been evaluated for the first time (Figs. 1, 2a). No histological alteration was observed in the LR apical meristem and in the primary differentiation zone of all genotypes untreated with As and/or Cd (Figs. 6a–c, 7a, h, n, Fig. S5). Also the treatment with 50 As did not induce notable cyto-histological alterations (data not shown). On the contrary, significant alterations were evident in the LR apical meristems of all genotypes after exposure to 60 Cd alone, 50 As plus 60 Cd and, but at a lesser extent, to 200 As (Fig. 6d–l). In particular, numerous and variously-sized vacuoles were present in the ground meristem cells of SR1, *rolB* and *rolB*-*AtPCS1* LRs after 60 Cd treatment (Fig. 6d–f and insets), with these cells losing their meristematic features concomitantly, and also showing reduced in size and multi-nucleolate nuclei (Fig. 6, inset in e). In the apical meristem of LRs of all genotypes, the initial cells of the endodermis appeared thin (Fig. 6d–f arrowheads). In all genotypes, all these cytological alterations were rare after exposure to 200 As, with the majority of the LRs resembling those of the Control treatment (Fig. 6g–i, a–c, in comparison). The exposure to 50 As + 60 Cd induced similar, even less severe, cytological alterations in comparison with the Cd alone treatment, because cell vacuolation was reduced (Fig. 6j–l, d–f, in comparison).

At the level of the primary structure zone, the LRs of all genotypes of the Control treatment showed a regular structure, both within and out of the stele (Fig. 7a, h, n, Fig. S5a–c). In the presence of 60 Cd, the SR1 roots showed cellular and nuclear hypertrophy in the cortex, and nucleolar fragmentation (Fig. 7b–c, arrows in c). An enhancement in hair formation also occurred in comparison with the LRs in the untreated plants (Fig. 7b, d). The cell walls of the cortical cells confining with the epidermis, i.e., the cells destined to become exodermis, were thickened by lignin deposition (Fig. S6a). The primary structure of SR1 LRs was similar in the presence of 50 and 200 As to that of the untreated LRs, except for a limited occurrence of nuclear hypertrophy in the cortex (Fig. 7e, a, in comparison). By contrast, the contemporary presence of As and Cd induced damages similar to those caused by Cd alone (Fig. 7f–g, b–c, in comparison). The treatment with 60 Cd alone also damaged the primary structure in LRs of *rolB* causing the same alterations as in the LRs cortex of SR1 (Fig. 7i, j, b), including cell wall thickening by lignin deposition in the external cortical cells (Fig. S6b). Exposure to 50 and 200 As did not cause structural alterations in comparison with the Control treatment (Fig. 7h, k). The presence of As + Cd affected the primary structure zone in the same way as Cd alone, repeating the behaviour of SR1 LRs (Fig. 7l–m, i–j). After 60 Cd exposure, the primary structure of *rolB*-*AtPCS1* LRs was altered as in the other genotypes, including lignin deposition in cortical cells (Fig. S6c). However, a crushing of the endodermis and stelar cells also occurred (Fig. 7o, p). The *rolB*-*AtPCS1* LRs responded to 50 and 200 As treatments as SR1 LRs (Fig. 7q, e). Again, the combined presence of As and Cd caused the same anomalies as under Cd alone, repeating the response of the other genotypes under the same treatment (Fig. 7r, s).

### Overexpression of *AtPCS1* inhibits Cd and As extrusion from leaf trichomes

**Fig. 8:**
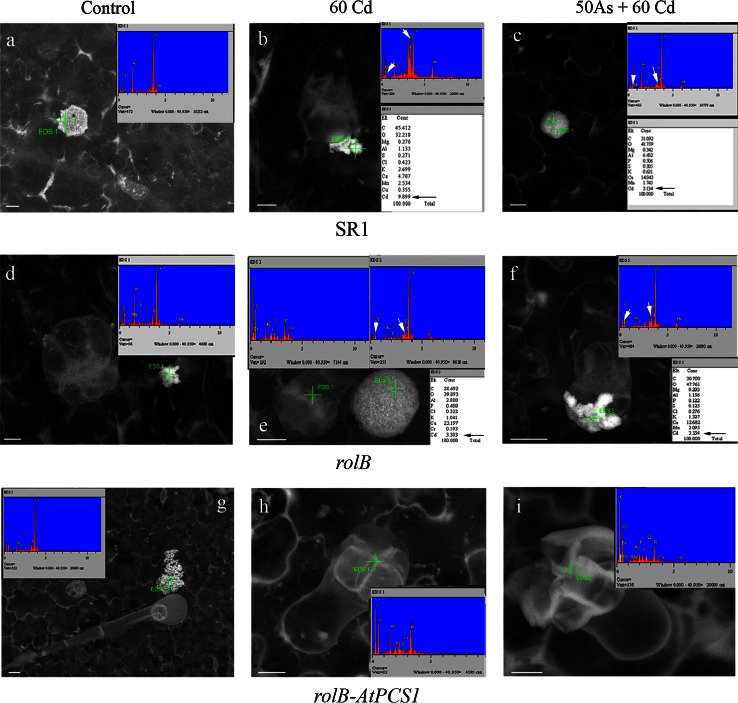
VP-SEM analysis on tobacco leaves showing crystal extrusion and chemical composition of the excreted crystals by EDS microanalysis (*insets*). Details of the leaf surface and trichomes of SR1 (**a**–**c**), *rolB* (**d**–**f**), and *rolB*-*AtPCS1* (**g**–**i**) plants grown for 5 weeks on refreshed MS medium without Cd, As (Control, **a**, **d**, **g**), or exposed either to 60 μM CdSO_4_ (60 Cd, **b**, **e**, **h**), or 50 μM Na_2_HAsO_4_.7H_2_O plus 60 μM CdSO_4_ (50 As + 60 Cd, **c**, **f**, **i**). Cadmium presence in the extruded crystals after exposure to Cd and Cd plus As is detailed in the *insets* of **b**, **c**, **e**, and **f** (*arrows*). The absence of Cd in the extruded crystals in the Control treatment for all genotypes, and in all treatments for *rolB*-*AtPCS1*, is shown by the *insets* in **a**, **d**, **g**, **h**, and **i**. *Bars* 10 µm (**b**, **d**, **i**), 20 µm (**a**, **c**, **e**, **f**, **g**, **h**)

Scanning electron microscopy analysis revealed that the head cells of the short trichomes on SR1, *rolB* and *rolB*-*AtPCS1* leaves extruded crystals, some of which remained observable on the leaf surface (Fig. 8). To determine the element composition of crystals, EDS analysis was performed on leaves of all genotypes not treated and treated with 60 Cd or 50 As plus 60 Cd for 2, 5, 6 and 8 weeks on refreshed MS medium. The period of exposure to the pollutants lasted more weeks, because after 2 weeks the extruded crystals did not contain Cd or As, independently of the genotype. Regardless to semimetal and metal exposure, the analysis showed that the main constituents of crystals were Ca, C, O followed by Al and Mg (Fig. 8 insets in a, d, g). After 5 weeks of 60 Cd exposure, only the crystals extruded by SR1 leaves contained Cd (Fig. 8b and inset). Another week was necessary to detect Cd in the crystals extruded by *rolB* leaves (Fig. 8e and inset). Cadmium was never observed in the crystals extruded by *rolB*-*AtPCS1* plants even after 8 weeks of Cd treatment (Fig. 8h and inset). The combined exposure to As and Cd delayed Cd extrusion from SR1 and *rolB* leaves of about 2–3 weeks (Fig. 8c, f and inset) and totally inhibited Cd extrusion from *rolB*-*AtPCS1* leaves (Fig. 8i and inset). Arsenic extrusion never occurred in all genotypes.

## Discussion

In the present work, we demonstrated that the *PCS1* gene from *Arabidopsis thaliana* overexpressed in *rolB*-expressing tobacco leads to significantly enhanced As and/or Cd accumulation in roots accompanied by an increased PC content and an increased capacity of detoxification of both pollutants.

### Higher PC levels, due to *AtPCS1* overexpression, are responsible of an enhanced As plus Cd detoxification

Present results demonstrate that the overexpression of *AtPCS1* in tobacco plants exposed to As and/or Cd, for 9 and 16 days, resulted in higher PC levels, both in the entire plants and in roots and shoots separately, when compared with SR1 and *rolB* plants (Fig. 3, Fig. S2), thus suggesting that these higher levels allow the sequestration of higher amounts of Cd and/or As. In accordance, PC accumulation, and increased detoxification, have been reported to occur in tobacco plants of the same *rolB*-*AtPCS1* line exposed for 9 days to CdSO_4_ alone (Pomponi et al. 2006), in *Brassica juncea* expressing *AtPCS1,* and in Arabidopsis expressing *Allium sativum PCS1*(*AsPCS1*) and Yest Cadmium Factor1 (*YCF1*), after treatment for 10–14 days with Na_2_AsO_4_ or CdCl_2_, respectively (Gasic and Korban 2007; Guo et al. 2012). In the present conditions, in all genotypes, after As and/or Cd exposure, endogenous GSH reached and maintained levels higher than in the Control treatment (Fig. 3, Fig. S2, insets). Similar results have been previously obtained in tobacco expressing *Ceratophyllum demersum PCS1* (*35S::CdPCS1*), exposed to either Cd or As and in Arabidopsis overexpressing *AtPCS1* exposed to Cd (Brunetti et al. 2011; Shukla et al. 2012). Taken together, the present increased detoxification of *rolB*-*AtPCS1* to As and/or Cd may be sustained by a GSH level maintained high over time. Wojas and co-workers (2010a, b) have reported that tobacco plants overexpressing *AtPCS1,* treated with CdCl_2_ or NaH_2_AsO_4_ for 2–3 days, show lower levels of PCs and endogenous GSH, compared to wild-type plants, and a reduced detoxification to the toxic elements. It is possible that this plant susceptibility is only initial, and that the exposure to the semimetal/metal for a longer period, as in the present research, might restore the cellular GSH/PC balance, and cell GSH homeostasis, finally resulting into an improved semimetal/metal detoxification.

The exposure to Cd, alone or combined with As, caused conspicuous cyto-histological damage in roots of all genotypes (Figs. 6, 7), nevertheless the *rolB*-*AtPCS1* plants showed higher detoxification in comparison with the other genotypes. This might be due to their higher Cd accumulation in the vacuoles, occurring after an exposure period long enough to allow the synthesis of the transporters of the PC-Cd complexes into the vacuole. It has been been demonstrated that chelation, followed by vacuolar compartmentalization, increases the tolerance and accumulation to Cd and As in Arabidopsis plants expressing both *AsPCS1* and *YCF1* (Guo et al. 2012). Accordingly, in our system, the increased levels of PCs, combined with the high levels of GSH (Fig. 3, Fig. S2), might be responsible of the chelation and vacuolar sequestration of Cd, but also of As. In fact, the semimetal is complexed and sequestered similarly to Cd (Song et al. 2010).

We found that PC3 was the dominant PC fraction in plants exposed to all treatments with Cd, whereas PC2 was the dominant fraction in plants exposed to As alone (Fig. 3, Fig. S2). These data are in accordance with those on tobacco plants overexpressing *AtPCS1* and exposed to Cd or As (Pomponi et al. 2006; Wojas et al. 2010a, b). In addition, the As-tolerant populations of *Holcus lanatus* and *Silene vulgaris* have been reported to produce higher concentrations of PC2 than the non-tolerant populations, which, on the contrary, synthesize mostly PC3 (Sneller et al. 1999; Hartley-Whitaker et al. 2001). We suppose that the presence of different PC fractions in our plants may be related, not only to the semimetal/metal detoxification (Schulz et al. 2008), but also to a higher toxicity of Cd in comparison with As.

### The simultaneous exposure to As and Cd modifies uptake, accumulation and detoxification, in comparison with the single element exposure

Results show that the combined presence of As and Cd enhances Cd accumulation in the roots, in particular of *AtPCS1* overexpressing plants, but reduces As accumulation, with respect to the same plants exposed to the single elements (Fig. 4, Fig. S4). In the mean time, the *AtPCS1* overexpressing plants showed improved FW and PR length in comparison with the other genotypes (Figs. 1, 2). In this work, arsenate salt (Na_2_HAsO_4_.7H_2_O) was used because it is the main As species in aerobic soils and enters root cells using transporters in competition with phosphate (Meharg and Hartley-Whitaker 2002). In addition, four genes encoding phosphate transporters are known in tobacco, among them *NtPT1* and *NtPT2* are strongly expressed in roots (Kai et al. 2002; Bucher 2007). These transporters might be used by As(V) to entry into the cells, and cause the very high level of As found in the roots after exposure to 200 µM Na_2_HAsO_4_.7H_2_O As, in particular.

Cadmium can easily enter into the root cell as Cd^2+^ mainly via cation (Ca^2+^, Fe^2+^, Mn^2+^) channels (Lindberg et al. 2004; Clemens 2006) and ZIP (Zinc regulated transporter/Iron-regulated transporter-like Protein) transporters (Lux et al. 2011 and references therein). We observed that the combined presence of As and Cd decreased As uptake, increasing Cd uptake (Fig. 4, Fig. S4). A reduction of As influx by suppressing phosphate/arsenate uptake systems has been reported to occur in plants in response to elevated concentrations of various pollutants (Meharg 1994). Similarly, the present reduction of As levels in plants of all genotypes exposed to As and Cd might result from a reduced activity of phosphate/arsenate uptake system. On the contrary, the higher Cd level in the roots of the same plants might be due to an easier uptake of the metal because of its high affinity with the numerous transporters/channels of essential bivalent cations. However, the combined exposure to Cd and As decreased Cd storage in the vacuoles with respect to Cd alone treatment (Fig. 5). This might be due to a competition between the complexes PC-Cd and PC-As for the same vacuolar transporters. In Arabidopsis, both PC-Cd and PC-As [i.e., PC-As(III)] complexes use the AtABCC1, AtABCC2 and AtABCC3 vacuolar transporters for accumulating either As or Cd in the vacuoles (Song et al. 2010; Park et al. 2012; Brunetti et al. 2015). In the hypothesis that similar transporters are active in tobacco, it is possible that a good part of Cd remains out of the vacuole, i.e., in the cytosol and cell wall structures.

We here show that *AtPCS1* overexpression does not promote Cd transport to the aerial organs (Fig. 4, Fig. S4) sustaining previous results about the absence of an increase of Cd root-to-shoot transport in tobacco overexpressing either *AtPCS1* or *OsACA6* (Pomponi et al. 2006; Shukla et al. 2014). When As and Cd were present in the root cells, the supposed competition for PC-complex formation between the two ions, and vacuole compartmentalization, resulted into an increased As transport to the aerial organs.

### *AtPCS1* overexpression does not reduce the cyto-histological damages in the roots

The exposure to Cd alone, or combined with As, was here shown to induce extensive damages to root tissues, even in the presence of *AtPCS1* overexpression, whereas the exposure to As alone caused a limited damage (Figs. 6, 7). In the root, the semimetal and metal, as the other ions, are transported radially from the rhizodermis, through the apoplast and/or symplast fluxes, across the cortex and the endodermis, up to the xylem in the stele. The roots may develop different types of anatomical barriers to limit the apoplasmic movement of toxic ions to the xylem. These barriers may be enhanced by the level of ion toxicity (Lux et al. 2011). In accordance, in the roots of tobacco plants exposed for 9 days to Cd or Cd plus As, we observed an increase in cell wall thickness, due to lignin over-deposition, in the rhizodermal and external cortical parenchyma cells of the primary structure zone, leading to a premature exodermis formation (Fig. 7, Fig. S6). This anomaly may be interpreted as a plant strategy to limit the access of the toxic metal, similarly to what is observed in other species after Cd exposure (Lux et al. 2011, and references therein). However, the formation of this barrier was not very efficient in protecting the plant from the Cd/As influx, because the high levels of the ions continued to be observed in its tissues up to day 16 (Fig. S4). In addition, we observed extensive vacuolization in meristematic cells (Fig. 6), as also reported in the root meristematic cells of *Allium cepa* after Cd treatment (Liu and Kottke 2004). Other cytological events frequently observed were nucleolar fragmentation, nuclear hypertrophy, and crushing of endodermal cells (Figs. 6, 7). These events are known to be caused by various stresses (Chen et al. 1988), including Cd response (Sanità di Toppi et al. 2012), and their occurrence indicates cell suffering. In our case, this suffering did not lead to cell/organ death.

The higher PC production in the *AtPCS1* overexpressing plants, enhancing the vacuolar Cd compartmentalization in the cortical parenchyma cells, in particular (Fig. 5), enhances the Cd sequestration in the root and increases the detoxification in the rest of the plant. Taken together the cyto-histological damage is used as a repair in the presence of *PCS1* overexpression.

### Overexpression of *AtPCS1* inhibits Cd and As detoxification through extrusion in leaf crystals

We observed that tobacco plants translocated a small amount of Cd in the aerial organs (Fig. 4, Fig. S4). Scanning electron microscopy and SEM–EDS analysis on tobacco leaves revealed that SR1 and *rolB* plants extruded crystals containing Cd, Ca and other elements (Fig. 8), in accordance with previous results in the same plant (Choi et al. 2001; Choi and Harada 2005). On the contrary, Cd was never observed in the crystals extruded by leaves of plants overexpressing *AtPCS1*. It has been suggested that in wild-type tobacco the production and active extrusion of crystals containing metals, such as Cd or Zn, is a way to eliminate the excess of toxic metals from the plant tissues (Choi et al. 2001; Sarret et al. 2006). In accordance with this hypothesis, we suggest that the presence of Cd in the crystals extruded from the leaves of SR1 and *rolB* plants can be a mechanism for metal detoxification in these plants. The absence of Cd-rich crystals on the leaves of *rolB*-*AtPCS1* plants cultured in the presence of Cd alone can be due to the higher Cd vacuolar accumulation in their leaves (Fig. 5s), not rendering necessary the metal extrusion from the leaf as an alternative detoxification mechanism. The absence of Cd in the crystals extruded from the leaves of *rolB*-*AtPCS1* plants cultured in the presence of Cd plus As might be differently interpreted, because there is no higher Cd vacuolar accumulation than in the leaves of SR1 and *rolB* (Fig. 5s). It is possible that the higher levels of PCs in the leaves of the overexpressing *AtPCS1* plants, chelating also As, cause a transport of the semimetal into the vacuole competitively reducing that of Cd. Thus, a part of Cd, remaining in the cytosol of the mesophyll cells as PC-Cd complex, might be translocated via phloem, again towards the root. In fact, it is known that the PC-Cd complexes are also involved in the long-distance shoot-to-root transport via phloem, both in *Arabidopsis*, and *Brassica* (Van Belleghem et al. 2007; Mendoza-Cózatl et al. 2011). The latter strategy would justify the absence of the Cd-leaf extrusion, and is consistent with the increased levels of Cd observed in the roots of *AtPCS1* overexpressing plants.

In conclusion, the overexpression of *AtPCS1* activates peculiar mechanisms, e.g., high levels of PCs and of Cd vacuolar accumulation, to reduce the toxicity of Cd and As in tobacco. The resulting increased detoxification capacity is important for further applications in phytoremediation. It is, in fact, possible that the detoxification mechanisms may be part of the acclimation process to the pollutants, necessarily involved in the acquisition of plant tolerance, and growth, in multi-contaminated environments.

#### *Author contribution statement*

ZL and FL designed and carried out the research. DAS and DRF contributed to the research. BP carried out molecular analyses. MC carried out the molecular analysis, contributed to the experimental design and provided tobacco transgenic lines. RE and LC carried out the SEM–EDS analyses on leaf crystals. BM carried out the ICP analyses. SdiTL and DF carried out the thiol peptide analyses. SL provided the know-how for Cd-fluorescence analysis. AMM and GF analyzed data and wrote the manuscript. All authors read and approved the manuscript.

## Electronic supplementary material

Fig. S1 Expression of *AtPCS1* gene in transgenic *rolB*–*AtPCS1* compared with *rolB* and SR1 tobacco plants. RT-PCR of total RNA extracted from 16-day-old seedlings grown in the absence of Cd/As. For all the reactions actin was run as an internal control (PDF 932 kb)

Fig. S2 Mean levels of PCs (PC2, PC3, PC4, PC5 fractions and total PCs) and of endogenous GSH (± SE) in SR1, *rolB* and *rolB*-*AtPCS1* plants grown on refreshed MS medium for 16 days either without Cd and As (Control, *insets*) or with either 50 or 200 μM Na_2_HAsO_4_.7H_2_O (50 As and 200 As, respectively), or 60 μM CdSO_4_ (60 Cd), or with 50 μM Na_2_HAsO_4_.7H_2_O plus 60 μM CdSO_4_ (50 As + 60 Cd). *Letter a*, *P* < 0.01 difference in the total PCs within the same treatment. *Letter b*, *P* < 0.05 difference in the total PCs with *rolB*-*AtPCS1* within the same treatment. Columns of total PCs followed by the *same letter/no letter*, within the same treatment, are not significantly different. Significant differences between different PC fractions and between treatments are reported in the text. Means of three replicates (PDF 1006 kb)

Fig. S3 Levels of As and Cd in roots and shoots of SR1, *rolB* and *rolB*-*AtPCS1* plants non-treated with Cd and As (Control treatment). Plants cultured for 9 days (**a**,**b**) and 16 days (**c**) either on Hoagland liquid medium (**a**,**c**) or on MS solid medium (**b**) after the growth for 10 days on MS germination medium (PDF 296 kb)

Fig. S4 Mean concentrations (± SE) of As and Cd in roots and shoots of SR1, *rolB* and *rolB*-*AtPCS1* plants treated for 16 days on Hoagland medium with either 50 or 200 μM Na_2_HAsO_4_·7H_2_O (50 As and 200 As, respectively), or 60 μM CdSO_4_ (60 Cd), or with 50 μM Na_2_HAsO_4_·7H_2_O plus 60 μM CdSO_4_ (50 As + 60 Cd) after the growth for 10 days on MS germination medium. *Letter a*, *P* < 0.01 difference within the same treatment. *Letter b*, *P* < 0.05 difference with *rolB*-*AtPCS1* within the same treatment. *Letter c*, *P* < 0.01 difference with *rolB*-*AtPCS1* within the same treatment. *Letter d*, *P* < 0.05 difference within the same treatment. Columns followed by the *same letter/no letter* within the same treatment are not significantly different. Significant differences between treatments are reported in the text. Means of three replicates (PDF 732 kb)

Fig. S5 Cross sections of regular primary structure in lateral roots of SR1 (**a**), *rolB* (**b**) and *rolB*-*AtPCS1* (**c**) non-treated with the semimetal/metal after 9 days of plant treatment on refreshed MS medium. *Bars* 30 µm (PDF 3228 kb)

Fig. S6 Cross sections of SR1 (**a**)*, rolB* (**b**) and *rolB*-*AtPCS1* (**c**) roots after 9 days of treatment with 60 μM CdSO_4_ on refreshed MS medium showing abundant lignin deposition, shown by the autofluorescence signal, in the cell walls of the exodermal cells. *Bars* 50 µm (PDF 2801 kb)

## Abbreviations

GSH: Tripeptide glutathione
LR: Lateral root
PC: Phytochelatin
PCS: Phytochelatin synthase
PR: Primary root
