# Supplementary material for: Overexpression of AtPCS1 in tobacco increases arsenic and arsenic plus cadmium accumulation and detoxification
Source: Planta. 2015 Nov 13;243:605–22. doi: 10.1007/s00425-015-2428-8 (PMC4757632; doi:10.1007/s00425-015-2428-8)
Supplement: Supplementary file 1 — Fig. S1 Expression of AtPCS1 gene in transgenic rolB–AtPCS1 compared with rolB and SR1 tobacco plants. RT-PCR of total RNA extracted from 16-day-old seedlings grown in the absence of Cd/As. For all the reactions actin was run as an internal control (PDF 932 kb) [file 425_2015_2428_MOESM1_ESM.pdf]

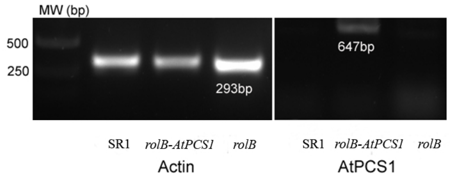

Supplementary Fig. S1

Article title: Overexpression of *AtPCS1* in tobacco increases Arsenic and Arsenic plus cadmium accumulation and detoxification

Journal name: Planta

Author names: Zanella L, Fattorini L., Brunetti P, Roccotiello E, Cornara L, D'Angeli S, Della Rovere F, Cardarelli M, Barbieri M, Sanità di Toppi L, Degola F, Lindberg S, Altamura MM, Falasca G.

Correspondign Author: Department of Environmental Biology, Sapienza University of Rome -Italy e-mail: giuseppina.falasca@uniroma1.it
