# Supplementary material for: Overexpression of AtPCS1 in tobacco increases arsenic and arsenic plus cadmium accumulation and detoxification
Source: Planta. 2015 Nov 13;243:605–22. doi: 10.1007/s00425-015-2428-8 (PMC4757632; doi:10.1007/s00425-015-2428-8)
Supplement: Supplementary file 2 — Fig. S2 Mean levels of PCs (PC2, PC3, PC4, PC5 fractions and total PCs) and of endogenous GSH (± SE) in SR1, rolB and rolB-AtPCS1 plants grown on refreshed MS medium for 16 days either without Cd and As (Control, insets) or with either 50 or 200 μM Na2HAsO4.7H2O (50 As and 200 As, respectively), or 60 μM CdSO4 (60 Cd), or with 50 μM Na2HAsO4.7H2O plus 60 μM CdSO4 (50 As + 60 Cd). Letter a, P < 0.01 difference in the total PCs within the same treatment. Letter b, P < 0.05 difference in the total PCs with rolB-AtPCS1 within the same treatment. Columns of total PCs followed by the same letter/no letter, within the same treatment, are not significantly different. Significant differences between different PC fractions and between treatments are reported in the text. Means of three replicates (PDF 1006 kb) [file 425_2015_2428_MOESM2_ESM.pdf]

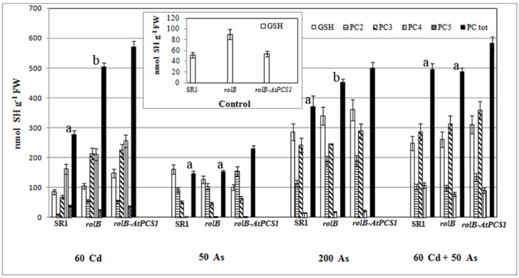

Supplementary Fig S2

Article title: Overexpression of *AtPCS1* in tobacco increases Arsenic and Arsenic plus cadmium accumulation and detoxification

Journal name: Planta

Author names: Zanella L, Fattorini L., Brunetti P, Roccotiello E, Cornara L, D’Angeli S, Della Rovere F, Cardarelli M, Barbieri M, Sanità di Toppi L, Degola F, Lindberg S, Altamura MM, Falasca G.

Correspondign Author: Department of Environmental Biology, Sapienza University of Rome -Italy e-mail: giuseppina.falasca@uniroma1.it
