# Supplementary material for: Overexpression of AtPCS1 in tobacco increases arsenic and arsenic plus cadmium accumulation and detoxification
Source: Planta. 2015 Nov 13;243:605–22. doi: 10.1007/s00425-015-2428-8 (PMC4757632; doi:10.1007/s00425-015-2428-8)
Supplement: Supplementary file 3 — Fig. S3 Levels of As and Cd in roots and shoots of SR1, rolB and rolB-AtPCS1 plants non-treated with Cd and As (Control treatment). Plants cultured for 9 days (a,b) and 16 days (c) either on Hoagland liquid medium (a,c) or on MS solid medium (b) after the growth for 10 days on MS germination medium (PDF 296 kb) [file 425_2015_2428_MOESM3_ESM.pdf]

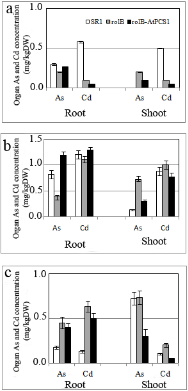

Supplementary Fig S3

Article title: Overexpression of *AtPCS1* in tobacco increases Arsenic and Arsenic plus cadmium accumulation and detoxification

Journal name: Planta

Author names: Zanella L, Fattorini L., Brunetti P, Roccotiello E, Cornara L, D'Angeli S, Della Rovere F, Cardarelli M, Barbieri M, Sanità di Toppi L, Degola F, Lindberg S, Altamura MM, Falasca G.

Correspondign Author: Department of Environmental Biology, Sapienza University of Rome -Italy e-mail: giuseppina.falasca@uniroma1.it
