# Supplementary material for: Overexpression of AtPCS1 in tobacco increases arsenic and arsenic plus cadmium accumulation and detoxification
Source: Planta. 2015 Nov 13;243:605–22. doi: 10.1007/s00425-015-2428-8 (PMC4757632; doi:10.1007/s00425-015-2428-8)
Supplement: Supplementary file 4 — Fig. S4 Mean concentrations (± SE) of As and Cd in roots and shoots of SR1, rolB and rolB-AtPCS1 plants treated for 16 days on Hoagland medium with either 50 or 200 μM Na2HAsO4·7H2O (50 As and 200 As, respectively), or 60 μM CdSO4 (60 Cd), or with 50 μM Na2HAsO4·7H2O plus 60 μM CdSO4 (50 As + 60 Cd) after the growth for 10 days on MS germination medium. Letter a, P < 0.01 difference within the same treatment. Letter b, P < 0.05 difference with rolB-AtPCS1 within the same treatment. Letter c, P < 0.01 difference with rolB-AtPCS1 within the same treatment. Letter d, P < 0.05 difference within the same treatment. Columns followed by the same letter/no letter within the same treatment are not significantly different. Significant differences between treatments are reported in the text. Means of three replicates (PDF 732 kb) [file 425_2015_2428_MOESM4_ESM.pdf]

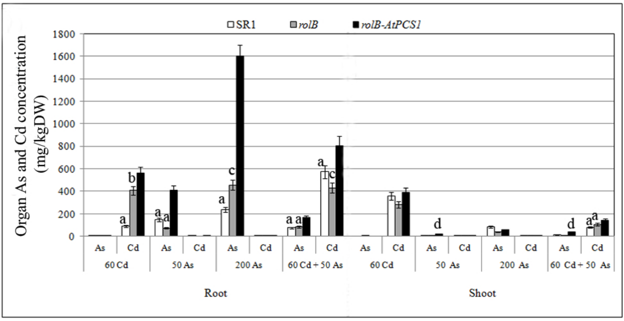


# Supplementary Fig S4

Article title: Overexpression of *AtPCS1* in tobacco increases Arsenic and Arsenic plus cadmium accumulation and detoxification

Journal name: Planta


Author names: Zanella L, Fattorini L., Brunetti P, Roccotiello E, Cornara L, D'Angeli S, Della Rovere F, Cardarelli M, Barbieri M, Sanità di Toppi L, Degola F, Lindberg S, Altamura MM, Falasca G.

Correspondign Author: Department of Environmental Biology, Sapienza University of Rome -Italy e-mail: giuseppina.falasca@uniroma1.it
