# Supplementary material for: Overexpression of AtPCS1 in tobacco increases arsenic and arsenic plus cadmium accumulation and detoxification
Source: Planta. 2015 Nov 13;243:605–22. doi: 10.1007/s00425-015-2428-8 (PMC4757632; doi:10.1007/s00425-015-2428-8)
Supplement: Supplementary file 5 — Fig. S5 Cross sections of regular primary structure in lateral roots of SR1 (a), rolB (b) and rolB-AtPCS1 (c) non-treated with the semimetal/metal after 9 days of plant treatment on refreshed MS medium. Bars 30 µm (PDF 3228 kb) [file 425_2015_2428_MOESM5_ESM.pdf]

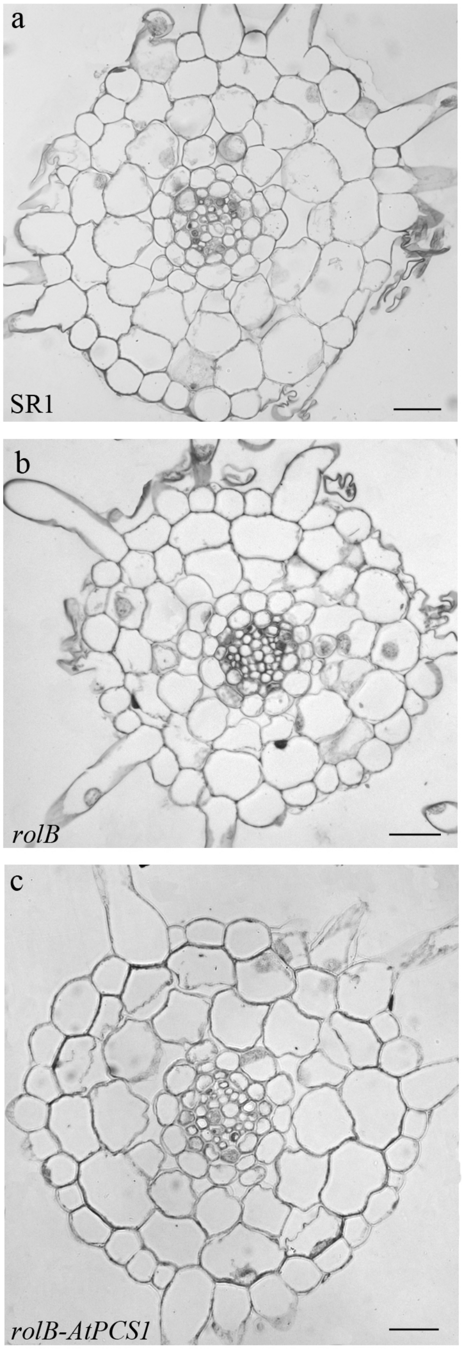

Supplementary Fig S 5

Article title: Overexpression of *AtPCS1* in tobacco increases Arsenic and Arsenic plus cadmium accumulation and detoxification

Journal name: Planta

Author names: Zanella L, fattorini L, Brunetti P, Roccotiello E, Cornara L, D’Angeli S, Della Rovere F, Cardarelli M, Barbieri M, Sanità di Toppi L, Degola F, Lindberg S, Altamura MM, Falasca G.

Corresponding Author: Department of Environmental Biology, Sapienza University of Rome - Italy, e-mail: giuseppina.falasca@uniroma1.it
