# Supplementary material for: Overexpression of AtPCS1 in tobacco increases arsenic and arsenic plus cadmium accumulation and detoxification
Source: Planta. 2015 Nov 13;243:605–22. doi: 10.1007/s00425-015-2428-8 (PMC4757632; doi:10.1007/s00425-015-2428-8)
Supplement: Supplementary file 6 — Fig. S6 Cross sections of SR1 (a), rolB (b) and rolB-AtPCS1 (c) roots after 9 days of treatment with 60 μM CdSO4 on refreshed MS medium showing abundant lignin deposition, shown by the autofluorescence signal, in the cell walls of the exodermal cells. Bars 50 µm (PDF 2801 kb) [file 425_2015_2428_MOESM6_ESM.pdf]

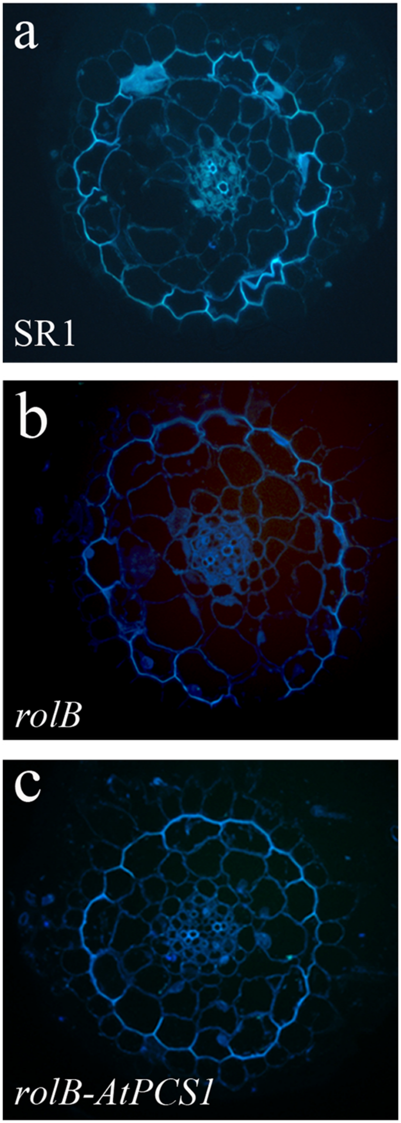

Supplementary Fig. S6

Article title: Overexpression of *AtPCS1* in tobacco increases Arsenic and Arsenic plus cadmium accumulation and detoxification

Journal name: Planta

Author names: Zanella L, Fattorini L., Brunetti P, Roccotiello E, Cornara L, D'Angeli S, Della Rovere F, Cardarelli M, Barbieri M, Sanità di Toppi L, Degola F, Lindberg S, Altamura MM, Falasca G.

Correspondign Author: Department of Environmental Biology, Sapienza University of Rome -Italy e-mail: giuseppina.falasca@uniroma1.it
